# SELF-MONITORING VIA WEARABLE SENSOR AND DAILY STEPS AMONG PEOPLE WITH MCI AND CARE PARTNER DYADS

**DOI:** 10.1093/geroni/igae098.1414

**Published:** 2024-12-31

**Authors:** Rashelle Hoffman, Blake Murphy, Vaishali Phatak, Diane Ehlers, Joan Monin

**Affiliations:** Creighton Univeristy, Creighton University, Nebraska, United States; Creighton University, Omaha, Nebraska, United States; University of Nebraska Medical Center, Omaha, Nebraska, United States; Mayo Clinic, Rochester, Minnesota, United States; Yale School of Public Health, New Haven, Connecticut, United States

## Abstract

Physical inactivity is a modifiable risk factor for Alzheimer’s Disease and Related Dementias (ADRD) that can be remediated using remotely delivered interventions. The 12-week telerehabilitation physical activity behavioral (TPAB) intervention includes receipt of a wearable sensor and weekly discussions about physical activity self-monitoring, goal setting, facilitators, and barriers. TPAB has been shown to be feasible and efficacious in increasing daily steps in older adults. However, evidence is lacking for TPAB’s use for people with MCI and using a dyadic approach (i.e., with their care partners). Results from the first phase of our study include descriptive information from two dyads about (1) dyad member interaction with the wearable sensor (i.e., how knowing the current daily step count impacted activity), (2) how dyad members helped each other overcome individual or shared barriers to daily stepping and (3) how dyad members planned activities together to achieve high daily average stepping. This project quantitatively identified (1) the highest stepping day each week was the same for both dyad members 46% of the time (2) the frequency of both dyad members meeting individual weekly stepping goals was 81% compared to just one dyad member or no dyad member reaching the weekly step goal. This project revealed barriers using wearable sensor technology (i.e., not looking at or wearing the wearable sensor). Our discussion will focus on how to enhance meaningful interaction with technology in dyads to track daily stepping that can be applied to improve behavioral change in people with MCI and care partners.

